# How protein targeting to primary plastids via the endomembrane system could have evolved? A new hypothesis based on phylogenetic studies

**DOI:** 10.1186/1745-6150-8-18

**Published:** 2013-07-11

**Authors:** Przemysław Gagat, Andrzej Bodył, Paweł Mackiewicz

**Affiliations:** 1 Department of Genomics, Faculty of Biotechnology, University of Wrocław, ul. Przybyszewskiego 63/77, Wrocław 51-148, Poland; 2 Laboratory of Evolutionary Protistology, Department of Evolutionary Biology and Ecology, University of Wrocław, ul. Przybyszewskiego 63/77, Wrocław 51-148, Poland

**Keywords:** Endomembrane system, Endosymbiont, Endoplasmic reticulum, Golgi apparatus, Horizontal gene transfer, Phylogeny, Plastid, Plastid transit peptide, Primary endosymbiosis, Protein trafficking, Signal peptide

## Abstract

**Background:**

It is commonly assumed that a heterotrophic ancestor of the supergroup Archaeplastida/Plantae engulfed a cyanobacterium that was transformed into a primary plastid; however, it is still unclear how nuclear-encoded proteins initially were imported into the new organelle. Most proteins targeted to primary plastids carry a transit peptide and are transported post-translationally using Toc and Tic translocons. There are, however, several proteins with N-terminal signal peptides that are directed to higher plant plastids in vesicles derived from the endomembrane system (ES). The existence of these proteins inspired a hypothesis that all nuclear-encoded, plastid-targeted proteins initially carried signal peptides and were targeted to the ancestral primary plastid via the host ES.

**Results:**

We present the first phylogenetic analyses of *Arabidopsis thaliana* α-carbonic anhydrase (CAH1), *Oryza sativa* nucleotide pyrophosphatase/phosphodiesterase (NPP1), and two *O. sativa* α-amylases (αAmy3, αAmy7), proteins that are directed to higher plant primary plastids via the ES. We also investigated protein disulfide isomerase (RB60) from the green alga *Chlamydomonas reinhardtii* because of its peculiar dual post- and co-translational targeting to both the plastid and ES. Our analyses show that these proteins all are of eukaryotic rather than cyanobacterial origin, and that their non-plastid homologs are equipped with signal peptides responsible for co-translational import into the host ES. Our results indicate that vesicular trafficking of proteins to primary plastids evolved long after the cyanobacterial endosymbiosis (possibly only in higher plants) to permit their glycosylation and/or transport to more than one cellular compartment.

**Conclusions:**

The proteins we analyzed are not relics of ES-mediated protein targeting to the ancestral primary plastid. Available data indicate that Toc- and Tic-based translocation dominated protein import into primary plastids from the beginning. Only a handful of host proteins, which already were targeted through the ES, later were adapted to reach the plastid via the vesicular trafficking. They represent a derived class of higher plant plastid-targeted proteins with an unusual evolutionary history.

**Reviewers:**

This article was reviewed by Prof. William Martin, Dr. Philippe Deschamps (nominated by Dr. Purificacion Lopez-Garcia) and Dr Simonetta Gribaldo.

## Background

Sometime prior to 1.5 billion years ago a phagotrophic eukaryote engulfed a cyanobacterium that was initially established as a permanent endosymbiont [1,2]. This process, called a primary endosymbiosis, eventually resulted in a primary plastid surrounded by two membranes. Descendants of this original plastid are present in three eukaryotic lineages comprising the supergroup Archaeplastida (formerly Plantae); these are the Glaucophyta, Rhodophyta and Viridiplantae, including green algae and land plants [3-6]. Transformation of the cyanobacterial endosymbiont into an integrated plastid involved two main processes: (i) gene transfer from the endosymbiont to the host nucleus, and (ii) the origin of translocons in the endosymbiont envelope to import proteins encoded by these nuclear genes [7-10]. It is estimated that modern primary plastids require at least 2,000 different proteins [11,12], whereas their genomes encode only between 60–200 [13]. The enormous difference in the number endosymbiont genes that reside in the nucleus, versus those retained in the plastid, reflects massive transfer (called endosymbiotic gene transfer, EGT) to the host nuclear genome, as well as loss of numerous bacterial genes that were no longer needed in a fully integrated organelle [14-17].

Most proteins imported into primary plastids carry an N-terminal targeting signal called a transit peptide (pTP) [18,19]. These proteins are recognized and moved post-translationally through the plastid envelope by two multi-subunit translocons in the outer (Toc) and inner (Tic) chloroplast membranes respectively [20-22]. These translocons consist of multiple specialized protein subunits that function as transit peptide receptors (Toc34, Toc64, and Toc159), protein-conducting channels (Toc75, Tic20, Tic21, and Tic110), regulatory elements (Tic32, Tic55, Tic62, and Tic40), and Toc-Tic translocon-connecting subunits (Toc12, Tic22).

Not all primary plastid-targeted proteins use the canonical Toc-Tic super-complex. For example, protochlorophylide oxidoreductase A (PORA) carries a transit peptide-like presequence [23] but is translocated across the outer plastid membrane through the OEP16 pore [24], which probably is derived from the mitochondrial Tim23 protein [8]. A more unusual import pathway is found in a small group of proteins targeted to higher plant plastids via the endomembrane system (ES), involving the endoplasmic reticulum (ER) and/or the Golgi apparatus. These proteins include α-carbonic anhydrase from *Arabidopsis thaliana* (CAH1) [25], as well as nucleotide pyrophosphatase/phosphodiesteras (NPP1) [26,27] and α-amylases αAmy3 [28] and αAmy7 [29,30] from *Oryza sativa*. They all carry N-terminal signal peptides (SP) responsible for their co-translational insertion into the ER. Apart from the signal peptide, CAH1 [25,31], NPP1 [26,27], and αAmy7 [29,30] also have complex glycan chains that direct their targeting to the plastid via the Golgi apparatus. An RNA-binding protein 60 (RB60) also was described from the green alga *Chlamydomonas reinhardtii* that is targeted to both the plastid and ER by means of a 50-amino acid N-terminal extension with characteristics of a typical signal peptide [32].

**Figure 1:**
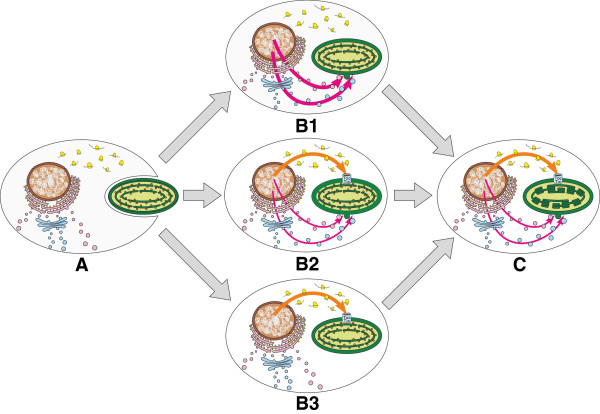
**Three evolutionary scenarios for the origin of endomembrane system-mediated protein targeting to higher plant plastids.** The phagotrophic ancestor of the kingdom Archaeplastida, including glaucophytes, red algae, and green plants, regularly fed on cyanobacteria, from which genes migrated to the host nucleus via endosymbiotic gene transfer **(A)**. When these endosymbionts evolved into primary plastids, they made use of both cyanobacteria- and host-derived genes present in the host nucleus. According to the ‘relic’ hypothesis for endomembrane system (ES)-mediated plastid protein targeting [33], all such proteins were targeted to the new primary plastid via the endoplasmic reticulum and/or Golgi apparatus **(B1)**. In a later evolutionary stage, this co-translational pathway was replaced by a post-translational route involving Toc and Tic translocons for most plastid-targeted proteins **(C)**. The hypothesis implies that proteins currently imported into higher plant plastids via the ES, such as αAmy3, αAmy7, CAH1, NPP1, are relics of ancestral ES-mediated protein trafficking to the primary plastid. Two alternative scenarios **(B2** and **B3)** conflict with the ‘relic’ hypothesis; they postulate that the Toc and Tic translocons evolved very early in the primary endosymbiosis. In one **(B2)**, a limited subset of host-derived proteins, previously targeted via the ES to different compartments within the host cell, exploited their pre-existing signal peptides to reach the primary plastid. Alternatively **(B3)**, host-derived proteins carrying signal peptides were directed to primary plastids much later, well after the initial primary endosymbiosis, and possibly only in some higher plant lineages **(C)**. Thickness of the colored arrows is proportional to the presumed or known commonality of a given pathway: ES (pink) or Toc-Tic translocons (orange). Stacked thylakoids only evolved in the green primary plastid lineage.

The unexpected discovery of proteins targeted to primary plastids through the ES inspired Bhattacharya and colleagues [33] to propose that, when endosymbiont genes first moved to host’s nucleus, they acquired signal peptides and were transported back to the ancestral primary plastid through the ER and/or Golgi apparatus (Figure 1 B1). Only later did Toc-Tic-based translocation machinery evolve and, because this new pathway was more efficient, selection favored modifications of signal peptides into transit peptides in hundreds of nuclear-encoded, plastid-targeted proteins. Consequently, almost all of them now use the Toc-Tic super-complex [33].

Based on this ‘relic’ hypothesis for ES-mediated plastid protein targeting [33], CAH1, NPP1, αAmy3, and αAmy7 would be a remnant of the ancestral ES-mediated protein targeting to primary plastids (Figure 1 B1); however, the evolutionary histories of these proteins have not been investigated previously. A cyanobacterial ancestry would be consistent with the hypothesis that they are relics of early endomembrane targeting. It would suggest that during their initial transfer to the host nucleus they acquired signal peptides, rather than transit peptides, consistent with the idea that ES targeting of proteins was present at the earliest stages of primary plastid evolution (Figure 1 B1). Alternatively, if proteins currently targeted to plastids via the ES have a host cell ancestry, it would suggest their ancestors carried signal peptides that targeted them, internally or externally, via the ES before the primary plastid endosymbiosis occurred. Here we present phylogenetic evidence that CAH1, NPP1, αAmy3, and αAmy7 are related to eukaryote-specific homologs that bear typical signal peptides and are trafficked through the host ES. Although redirection of these proteins to the plastid could have occurred during or shortly after the primary endosymbiosis (Figure 1 B2), our results indicate their targeting to plastids evolved only in the higher plant lineage, long after primary plastids were established (Figure 1 B3).

## Results

### α-Amylases

**Figure 2:**
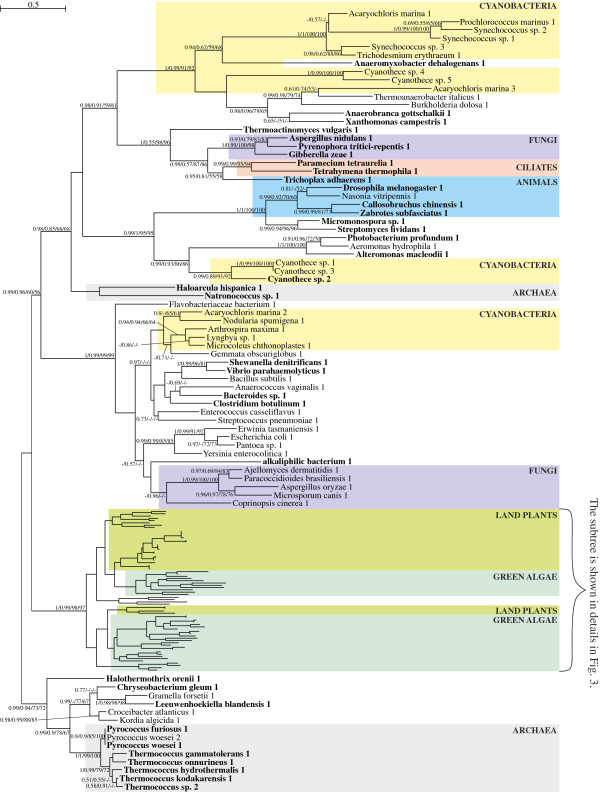
**The Bayesian tree for α-amylases obtained in PhyloBayes under the LG + Γ(5) model.** Sequence in which more than 50% algorithms recognized SP are indicated in bold. Numbers at nodes, in the presented order, correspond respectively to posterior probabilities estimated in PhyloBayes for LG + Γ(5) model (PP-LG) and CAT + Γ(5) model (PP-CAT), as well as support values resulting from bootstrap analysis in PhyMl (B-Ph) and TreeFinder (B-TF). Values of the posterior probabilities and bootstrap percentages lower than 0.5 and 50% were omitted or indicated by a dash “-“. All bacterial sequences, apart from cyanobacterial ones, are in white background.

Α-amylases, like αAmy3 and αAmy7 from *Oryza sativa,* are widely distributed in animals, plants, fungi, bacteria, and archaeans (Figure 2) [34-36]. They catalyze the hydrolysis of α-1,4 glycosidic bonds, but differ in substrate specificity; αAmy3 shows higher reactivity with oligosaccharides, whereas αAmy7 targets soluble starch and starch granules [37,38]. Both enzymes are active in the starchy endosperm in germinating seeds where they play crucial roles in starch degradation and seed germination [39]. Expression and secretion of both proteins are similar in the aleurone layer, but there are unique time- and tissue-specific expression patterns in the embryo [39].

Until recently, plastid localization of α-amylases was rather speculative. Some experiments based on subcellular fractionation, substrate-specific activities, and end-product analyses indicated this location [40-43], while others did not [44-46]. Thanks to additional and comprehensive investigations of αAmy3 [28] and αAmy7 [29,30], it is now certain that they are targeted to both primary plastids and the external cell wall matrix via the ES; αAmy7 differs from αAmy3 by the presence of an N-linked oligosaccharide side chain, which results in its trafficking through the Golgi apparatus [29,30,47]. It is possible that other amylases are targeted to plastids via the Golgi apparatus as well, because inhibition of Golgi secretion using brefeldin A results in dramatically increased starch accumulation in *Arabidopsis,* tobacco, and *Chlamydomonas* plastids [48].

**Figure 3:**
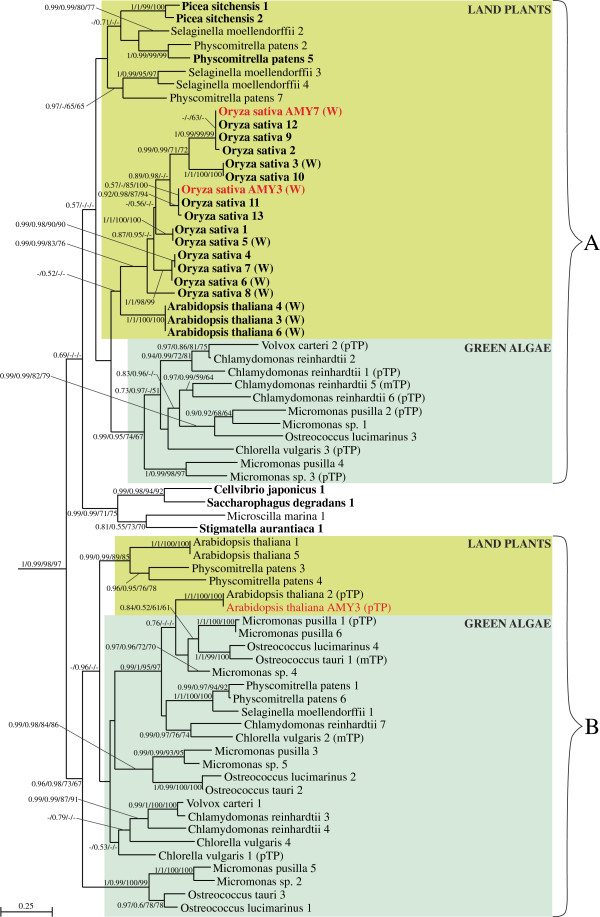
**Part of the Bayesian tree from Figure **2**for α-amylases of green algae and land plants obtained in PhyloBayes under the LG + Γ(5) model.** Sequences experimentally proved to be imported to plastids are in red font. Sequence in which more than 50% algorithms recognized signal peptide are indicated in bold and those which possess plastid transit peptide or mitochondrial transit peptide are signed respectively (pTP) and (mTP). Proteins that were shown to be located in the cell wall by mass spectrometry are indicated by (W). Other explanations as in Figure 2.

Phylogenetic trees of α-amylases produced by different methods show congruent topologies (Figures 2 and 3), and indicate that α-amylases were acquired independently by various groups of eukaryotes from bacteria via horizontal gene transfer (HGT) (Figure 2). There are at least four such eukaryotic clades scattered among bacteria that clearly are separated from each other. Respectively, these clades contain sequences from (i) ciliates, fungi, and placozoans, (ii) insects, (iii) fungi, and (iv) green plants. It is important to note, however, that green plant α-amylases are not of cyanobacterial origin; that is, they do not group together in our phylogenetic analyses (Figures 2 and 3). Moreover, alternative tree topologies that assume monophyly of plant and cyanobacterial sequences all are rejected with high confidence by all tests applied (see Methods).

Green plant α-amylases form two distinct clades, designated as A and B on the tree presented in Figure 3, and each includes both green algae and land plants. Interestingly, nearly all land plant sequences in clade A carry an N-terminal signal peptide and some of them have already been localized by mass spectrometry analyses to the cell wall [49]; both indicate ES-mediated targeting. This clade contains both *O. sativa* αAmy3 and αAmy7, which have been shown experimentally to be targeted to both the cell wall and plastid via the ES [28-30]. Only two enzymes, both from the spike-moss *Selaginella moellendorffii* (denoted 2 and 4 in Figure 3), were predicted to lack a signal peptide by more than 75% algorithms. All other sequences missing a signal peptide appear to be incomplete, based on the absence of an N-terminal methionine and, therefore, could carry targeting signals.

Interestingly, green algal proteins in clade A, and all green algal and land plant proteins in the clade B, were predicted to possess plastid or mitochondrial transit peptides rather than signal peptides. What is more, one of the proteins from the clade B with clearly predicted plastid transit peptide, αAmy3 from *A. thaliana*, was shown experimentally to be targeted to primary plastids [50]. The finding that enzymes with signal peptides are present only in land plant sequences clustered in the clade A, and that they nest within a broader family of α-amylases with transit peptides, all suggest that α-amylases initially were targeted to the green plastids via the Toc-Tic super-complex, and that signal peptides evolved later only in some land plant lineages.

It is interesting that starch metabolism, including α-amylase activity, appears to have been relocated to primary plastids relatively recently in the evolution of the Archaeplastida [51-54]. Recent molecular phylogenetic analyses by Ball and co-workers studies, along with earlier phycological studies from 1970s and 1980s [55-59], indicate that starch breakdown and synthesis occurred in the host cytosol in the common ancestor of glaucophytes, red algae, and green plants, and only was relocated to the plastid in green plants [51-54]. Genes involved in starch metabolism could have been transferred from the cyanobacterial to the host genome very early, possibly before an efficient plastid protein targeting system was established. These genes, in cooperation with eukaryotic enzymes could have helped to convert pre-existing cytosolic glycogen pathways into those for cytosolic starch synthesis [51-54]. Starch metabolism moved to the plastid only in green plants after they diverged from red algae, perhaps some 800 to 1,000 million years after the primary endosymbiosis [60,61]. According to the idea of ‘minor mis-targeting’ [62], the entire starch pathway could have been relocated to the ancestral green plastid in a single step; however, available data suggest that the relocation proceeded in a step-by-step manner, from synthesis of small pools of unbranched malto-oligosaccharides through glycogen and finally to starch [51]. The force that drove starch metabolism relocation was likely the appearance of novel light-harvesting antennae in plastids, and the concurrent demand for energy to reduce photo-oxidative stresses associated with their evolution [51,52].

We suggest that the emergence of α-amylases with signal peptides in land plants (clade A) could be related to apoplastic secretion of these enzymes to nutritive tissues, such as the starchy endosperm of seed plants, to utilize oligosaccharides stored there. The increased need for efficient starch hydrolysis in higher plant plastids could have favored targeting additional α-amylases to these organelles. Since these signal peptide-carrying α-amylases did not lose their original metabolic functions and, consequently, still required glycosylation and/or are transport to the cell wall, they were redirected to plastids through their pre-existing endomembrane pathway.

In addition to the horizontal acquisitions of α-amylases mentioned previously, our phylogenetic analyses identified one more such transfer from green plants to bacteria. Specifically, we recovered three proteobacteria species, *Saccharophagus degradans*, *Cellvibrio japonicus*, *Stigmatella aurantica*, and one bacteroidete species, *Microscilla marina*, nested among green plant sequences with strong statistical support (Figure 3). The hypothesized HGT is reasonable, especially given aspects of the ecology of these bacteria. *Saccharophagus degradans*[63] and *M. marina*[64] were isolated from the marine environment, *C. japonicus* from soil [65], and *S. aurantica* from plant detritus [66]. The three proteobacterial species are known for their ability to degrade plant cell wall polysaccharides, whereas the bacteroidete species for remineralizating organic compounds of marine phytoplankton and detritus particles [64].

### Purple acid phosphatases

**Figure 4:**
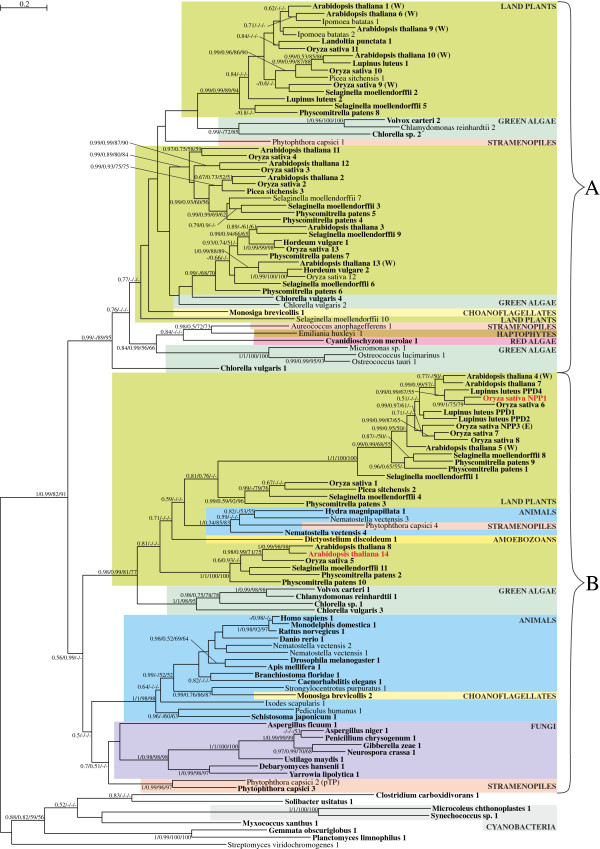
**The Bayesian tree for purple acid phosphatases obtained in PhyloBayes under the LG + Γ(5) model.** Sequences experimentally proved to be imported to plastids are in red font. Proteins that were shown to be located in the cell wall by mass spectrometry are indicated by (W), and those that were experimentally proved to reside in endomembrane system by (E). Other explanations as in Figure 2.

*Oryza sativa* NPP1 belongs to a family of purple acid phosphatases (PAPs) widely distributed in plants and animals, and also found in some fungi and bacteria (Figure 4) [67-69]. All PAPs are tartrate resistant metalloproteins that hydrolyze phosphate esters and anhydrides under acidic condition. Their active sites contain two metal ions, one is iron and the second iron, manganese, or zinc [67,70-73]. Because ferric compounds are oxidized, concentrated solutions of PAPs turn pink/purple, which is why they are called purple acid phosphatases [67,70-73]. NPP1 appears to be a member of a novel class of PAPs, along with diphosphonucleotide phosphatase/phosphodiesterases PPD1-4 characterized in *Lupinus luteus*[26,67,74]. They all share high sequence similarity but differ in substrate specificity [26,67,74].

Eukaryotic PAPs are secretory N-glycoproteins bearing signal peptides [67], and the sugar chains are necessary for enzymatic activity in at least some PAPs [75]. Mammalian enzymes possess mannose-6-phosphate that is specifically added in the *cis*-Golgi apparatus and targets proteins to lysosomes [76]. A similar pattern of high mannose glycosylation was observed in an extracellular PAP from the fungus *Aspergillus ficuum*[77]. Plant enzymes are mostly soluble secretory proteins [67] but, in addition to extracellular localization, they also are found in vacuoles [78], plastids [26], attached to the cell membrane [79], and are predicted to be targeted to mitochondria [68]. There probably are some ER membrane-anchored forms as well [67,74].

In accordance with their secretory nature, signal peptides are predicted in over 75% of PAPs in our analysis (96/119) by more than half the algorithms we employed (Figure 4). Only the sequence from the oomycete *Phytophthora capsici* (denoted with 2) was predicted to have a plastid transit peptide by three of five programs (the other two indicated a mitochondrial transit peptide). Among the PAPs analyzed, ten sequences had incomplete N-termini, meaning their complete genes could encode signal peptides. Because the secretory nature and tendency to be glycosylated are immanent features of purple acid phosphatases, it is not surprising that NPP1 from *O. sativa* also is a glycoprotein carrying a signal peptide (Figure 4) [26].

According to previous molecular phylogenetic studies, PAPs constitute an ancient protein family that existed before the split of plants, fungi, and animals [68,69,80]. Our phylogenetic analyses confirm this finding; we recovered two main eukaryotic clades, designated A and B, which are distinctly separated from bacteria (Figure 4). Alternative tree topologies assuming a cyanobacterial origin of PAPs in photosynthetic eukaryotes, including green plants, all were significantly worse than the best tree (see Methods). Clade A consists of sequences from photosynthetic eukaryotes (red algae, green algae, land plants, stramenopiles, and a haptophyte), the oomycete *P. capsici*, and one representative of choanoflagellates (*Monosiga brevicolis*). Based on our trees, both *M. brevicolis* and *P. capsici* probably acquired their PAP genes from green plants via HGT. *Phytophtora* species are parasites of higher plants [81] and *Monosiga* is a phagotrophic protozoan known to have taken up a variety of algal genes (for more examples, see [82]). Clade B comprises sequences from opisthokonts (choanoflagellates, fungi, and animals), the oomycete *P. capsici*, green green algae and land plants; it also contains *O. sativa* NPP1). The green lineage forms a distinct group that also includes a sequence from *P. capsici*, one from the slime mold *Dictyostelium discoideum* and two from the cnidarians *Nematostella* and *Hydra* (see Figure 4). The well-supported position of these non-photosynthetic eukaryotes within green plants suggests their PAPs were acquired by HGT.

The sub-clade including the plastid-targeted *O. sativa* NPP1 is sister to one containing NPP3, the other experimentally characterized protein from *O. sativa*[27]; however, NPP3 has been found only in cellular compartments outside the plastid, probably within the endomembrane system. What is more, another protein from *A. thaliana* (AT4G24890, denoted with 4 in Figure 4) is a closer homolog to NPP1 than to NPP3 and was localized to the cell wall by mass spectrometry [83,84]. In addition, *A. thaliana* AT1G13750 (denoted with 5 in Figure 4) is recovered as basal to *O. sativa* NPP1, NPP3, and *A. thaliana* AT4G24890. The AT1G13750 protein also was identified in the cell wall glycoproteome through mass spectrometry and chromatography [84,85]. Because the closest homologs to NPP1 are found outside the plastid, including the cell wall, we suggest that the ES-mediated targeting to the plastid evolved within the PAP family, and only in NPP1 from *O. sativa* (and perhaps related land plant orthologs that have yet to be identified).

Mass spectrometry showed that AT1G13900, another PAP from *A. thaliana* (denoted with 14 in Figure 4), is present in the plastid [86] and all the computational algorithms we applied indicate a signal peptide is present in its sequence. Therefore, this protein also could be targeted to the plastid via the ES; however, additional mass spectrometry analyses did not confirm plastid localization [87]. If further investigation of this protein verifies plastid targeting, its distant position from NPP1 on our tree (see Figure 4) would indicate that the proteins were redirected to higher plant plastids independently. Regardless of what further investigations of AT1G13900 reveal, our analyses strongly support an ancestor of *O. sativa* NPP1 that was a secretory protein targeted to the cell wall. Only later in evolution it was redirected to the plastid. Because, like αAMY7, NPP1 requires glycosylation, the pre-existing endomembrane pathway through the Golgi apparatus needed to be retained for this purpose.

### α-Carbonic anhydrases

*Arabidopsis thaliana* CAH1 belongs to a large family of α-carbonic anhydrases that is part of a larger assemblage including β, γ, δ, and ζ families [88]. Although these families share no significant sequence identity or structural similarity, they are all metalloenzymes (predominantly containing zinc) that catalyze the reversible hydration of carbon dioxide. This reaction not only allows the cell to concentrate CO_2_ at levels required for cellular enzymes, but also helps generally to maintain proper intracellular concentrations of CO_2_ and HCO_3_^‐^[89,90]. Carbonic anhydrases have evolved multiple times and, therefore, are an example of convergent evolution of catalytic function. Their metabolic significance is emphasized by the fact that they are found in all domains of life [88,90-92].

We performed phylogenetic analyses for CAH1 as discussed above for other ES-targeted plastid proteins. Unfortunately, the phylogenetic signal present in α-carbonic anhydrases is very weak and trees were poorly resolved (data not shown). Therefore, we had to examine the evolutionary history of the *Arabidopsis* CAH1 through alternative, careful consideration of available data.

The vesicular pathway of CAH1 to higher plant plastids appears to be an exception rather than the rule within this protein family. For example, nectarin III (NEC3), an α-carbonic anhydrase from tobacco, is a secretory protein present in nectar [93]. *NEC3* is expressed most strongly in the nectary gland and, at lower levels, in various floral organs. Its expression was not detected in leaves, which suggests it is not present in plastids nor important in plastid function [93]. The other member of α-carbonic anhydrase family is dioscorin, the major storage protein in yams (*Dioscorea* sp.) [94,95]. Immunolocalization showed it to be present in vacuoles rather than plastids of *Dioscorea* tuber cells [96].

Periplasmic and/or extracellular α-carbonic anhydrases were identified in the green algae *C. reinhardtii*[97-101], *Chlorella sorokiniana*[102], and *Dunaliella salina*[103]. Detailed studies definitely excluded that these enzymes are plastidial or cytoplasmic [97-103]. Periplasmic α-carbonic anhydrase activity also was reported in many other algae [104], indicating it could be very common and reflect the ancestral state. In agreement with a periplasmic localization, signal peptides clearly are present at their N-termini [97,100,102,103].

Plastid localization of α-carbonic anhydrases in green algae only has been reported for CAH3 from *C. reinhardtii*[105]. Unlike *Arabidopsis* CAH1 [25], the CAH3 presequence in *Chlamydomonas* does not contain an N-terminal signal peptide but, rather, a bipartite leader sequence composed of the classic plastid transit peptide followed by a signal peptide-like domain that functions as a thylakoid import signal. Localization of CAH3 in the thylakoid lumen was confirmed by immunoblot analyses and it is clear that this protein is not targeted to the plastid via the ES [105]*.*

To date, there are no firm data supporting ES-mediated trafficking of any α-carbonic anhydrases to higher plant plastids, other than CAH1 from *A. thaliana*[25]*.* Moreover, the main anhydrases present in plastids belong to the separate β family and are synthesized as precursor proteins carrying classic plastid transit peptides [104,106,107]. Thus, available data clearly indicate that CAH1 from *Arabidopsis* was adapted independently and secondarily to a plastid function, similar to the other proteins discussed above.

### Protein disulfide isomerases

**Figure 5:**
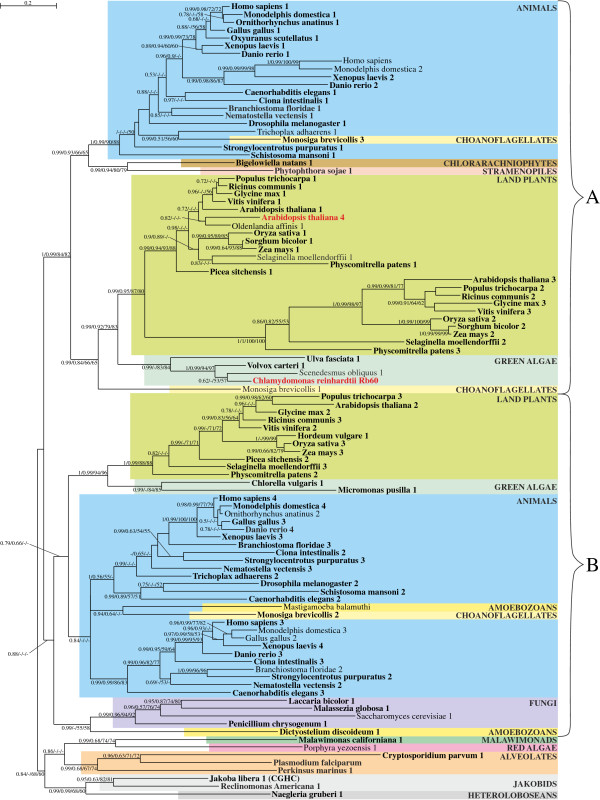
**The Bayesian tree for protein disulfide isomerases obtained in PhyloBayes under the LG + Γ(5) model.** Sequences experimentally proved to be imported to plastids are in red font. Other explanations as in Figure 2.

*Chlamydomonas reinhardtii* RB60 belongs to a family of protein disulfide isomerases (PDIs) that is widespread in eukaryotes, including plants, fungi, and animals (Figure 5) [108-110]. Prokaryotes have a functionally equivalent family of disulfide bond (Dsb) proteins [111]. PDIs catalyze formation, breakdown, and rearrangement of disulfide bonds, and also exhibit chaperone-like activity [108-110]. Like many other protein-folding factors, they usually are targeted to the ER by means of a signal peptide and are kept there by an ER retention signal [112]; however, some mammalian enzymes also have been found in the extracellular space, at the cell surface, in the cytosol, and in the nucleus [113].

Interestingly, dual ER and plastid localization was described for *Chlamydomonas* RB60 [32]. If the RB60 signal peptide is recognized by SRP (signal recognition particle), it is targeted to the ER where it remains thanks to its C-terminal KDEL motif, the ER-retention signal. If, however, NAC (nascent polypeptide associated complex) instead binds to its presequence, RB60 bypasses the ER and is imported post-translationally into the plastid through the Toc and Tic translocons [32]. Similar targeting patterns could be present in higher plants because a homolog of RB60 was recently identified in *Arabidopsis* plastids (denoted with 4 in Figure 5) [114-116]. The sequence of the higher plant homolog is equipped with a signal peptide and KDEL motif, indicating it also is retained within the ER.

In plastids, RB60 is involved in light-regulated translation of mRNA from *psbA*, which encodes the D1 protein for the photosystem II reaction centre complex [32]; its role in the ER is unknown and requires further investigation. Because *RB60* is the only PDI gene identified in *C. reinhardtii*, it could be responsible for other typical PDI reactions like disulfide oxidation, reduction, and rearrangement [32].

Because PDIs are not phylogenetically related to their bacterial analogs in the Dsb family [111], plastid PDIs cannot be of cyanobacterial origin; rather, they must have come from the eukaryotic host. Phylogenetic trees recovered using different methods show similar topologies with two large eukaryotic clades, designated as A and B, and a third small one containing excavates, alveolates, an amoebozoan, and a red alga (Figure 5). All other sequences from photosynthetic eukaryotes, mostly from green plants, form two distinct groups, one in each respective major clade. In addition to green plant sequences, clade A includes sequences from stramenopiles, chlorarachniophytes, choanoflagellates, and animals whereas clade B from amoebozoans, fungi, choanoflagellates, and animals. *Chlamydomonas* RB60 is located among other green algal proteins in clade A, basal to land plant sequences including the plastid homolog from *A. thaliana* (denoted with 4 in Figure 5). Another interesting feature of the PDI phylogeny is the placement of a sequence from the choanoflagellate *M. brevicolis* within green plants, suggesting HGT into this heterotrophic protist (see also [82,117,118]).

The peculiar targeting of RB60 could be considered a relic from ES-mediated transport to primary plastids especially, given its dual targeting and the presence of a homolog in higher plants [32,114-116]. The existence of an ER retention signal in this protein [112], however, argues against that evolutionary scenario. This motif prevents an ER-resident protein from being transported to additional locations within the ES; in the case of RB60, this would include the outer membrane of primary plastids. It suggests that PDIs never have been targeted to primary plastids via the ES. Such targeting could have happened only if *PDI* genes were duplicated and one copy had lost the ER retention signal. Only the latter copy could have exploited the endomembrane pathway to reach the plastid; however, there is no evidence for such a hypothetical duplication, and only one PDI is present in *C. reinhardtii* in any case. Moreover, all complete PDI sequences analyzed from green algae and higher plants (including all paralogs in *A. thaliana*) are equipped with the KDEL or its variant RDEL signals. This indicates that none of them should leave the ER to enter other compartments of the ES.

The general presence of PDI proteins in the ER in representatives of many different eukaryotic groups strongly supports such a localization in a common eukaryotic ancestor, and suggests that homologs of plastid PDIs originally also were targeted to this compartment. Redirection of *Chlamydomonas* RB60 to the plastid, and most likely the independent redirection of its homolog in *A. thaliana*, must proceed post-translationally, bypassing the ES, to avoid being trapped by the ER retention signal.

## Discussion

### Weaknesses of the ‘relic’ hypothesis for endomembrane system-mediated plastid protein targeting

It is commonly accepted that the two membrane-bound plastids of glaucophytes, red algae, and green plants evolved from a cyanobacterial endosymbiont via primary endosymbiosis [3-6]; however, their original method of importing nuclear-encoded proteins is still hotly debated [8-10,33,119]. This is largely because import involves distinct routes, each containing specific targeting signals. The large majority of proteins imported into primary plastids carry transit peptides and are moved through the two-membrane plastid envelope post-translationally via Toc and Tic translocons [9,20-22]. There are, however, some plastid proteins in higher plants, including αAmy3, αAmy7, NPP1, and CAH1, which are equipped with signal peptides and are co-translationally targeted to plastids through the ER and/or Golgi apparatus [25-30].

According to the ‘relic’ hypothesis for ES-mediated plastid protein targeting advanced by Bhattacharya and colleagues [33], the signal peptide-carrying plastid proteins are relics of ancestral ES-mediated protein targeting to primary plastids. During a hypothetical early evolutionary stage, all plastid-directed proteins derived from both the endosymbiont and the host would have been targeted to the ancestral primary plastid only via the ES. This hypotheses appears to overcome presumed difficulties with the rapid origin of a complicated, multisubunit Toc-Tic-based import apparatus [33]; however, upon further consideration, it is much less probable for several reasons. First, although modern Toc and Tic translocons are complex structures [20,22], initially they could have been as simple as single cyanobacterium-derived proteins functioning as protein-conducting channels [9]. For example, the main pore of the Toc complex, Toc75, not only contains a channel domain but also a receptor domain for transit peptides of imported proteins [120]. Originally, it could have inserted proteins into the outer plastid membrane by itself [121,122]. Second, the ‘relic’ hypothesis is relatively unparsimonious, postulating first the evolution of complex targeting signals composed of a signal peptide and internal sorting signals [27,30] in hundreds of nuclear-encoded, plastid-targeted proteins, followed by their later replacement by transit peptides, independently, in all these same proteins [9]. Moreover, there is no obvious selective advantage for such a traumatic transformation, which would have resulted in mis-targeting of numerous plastid proteins [9].

An additional argument against the ‘relic’ hypothesis comes from how protein transport occurs in eukaryotic alga-derived plastids in many eukaryotic lineages such as euglenids, cryptophytes, stramenopiles, dinoflagellates, and even parasitic apicomplexans [3-6]. Since all their nuclear-encoded proteins carry signal peptides and are targeted via the ES [123,124], they could be used to model early stages in the evolution of protein import in primary plastids. Most tellingly, no co-translational import pathway ever has been transformed into a post-translational system in any of these numerous lineages [9]. Two independent import routes for nuclear-encoded, plastid-targeted proteins have evolved in both euglenids and dinoflagellates, but all still proceed via the ES [125,126].

### Host origin of proteins targeted to higher plant plastids via the endomembrane system

All the arguments presented in the previous section encouraged us to test the ‘relic’ hypothesis formally. To determine whether ES-mediated transport of αAmy3, αAmy7, NPP1, and CAH1 could be left over from an ancestral stage of protein trafficking to primary plastids, we performed phylogenetic analyses on all four sequences. Our results, along with auxiliary data discussed in the results section, clearly show that none of genes encoding these proteins is cyanobacterial in origin. Rather, they all are derived from host cell sequences, excluding the possibility that they were transferred to the host cell from the cyanobacterial endosymbiont. Moreover, based on phylogenetic analyses, αAmy3, αAmy7, and NPP1 all are derived from homologs that contain signal peptides. We therefore suggest that αAmy3, αAmy7, NPP1, and CAH1 were pre-adapted to be delivered to primary plastids via the ES because their ancestral proteins carried signal peptides and were targeted to distinct compartments within the host ES. Thus, the signal peptide-carrying plastid proteins appear to represent a distinct class of proteins imported into modern primary plastids with a peculiar, but more derived evolutionary history.

Our model of retargeting of host proteins to higher plant plastids is further supported by the chimeric nature of the plastid proteome. According to the early ‘product-specificity corollary’ hypothesis, formulated by Weeden in 1981 [127], proteins targeted to primary plastids should be derived only from the cyanobacterial endosymbiont. This hypothesis was refuted by careful studies of evolutionary histories of higher plant enzymes involved in the Calvin cycle, which showed their mixed endosymbiotic and host origin [128,129], and further by analyses of the Toc and Tic translocons [22], the shikimate pathway for amino acid biosynthesis [130], and the oxidative pentose phosphate pathway [128]. Targeting of host proteins to primary plastids usually is accompanied by loss of homologous plastid-residing genes, a process known as endosymbiotic gene replacement [128,131-133]. In some cases, however, no homologs to host-derived plastid proteins were present in the original cyanobacterial endosymbiont, resulting in expansion of the plastid proteome [22,134,135]. It will be interesting to determine through further analyses which of these two possibilities is relevant to αAmy3, αAmy7, CAH1, and NPP1. αAmy3 and αAmy7, in particular, represent a peculiar case. In the ancestor of the Archaeplastida, it appears that starch metabolism was relocated from the cyanobacterial endosymbiont to the host cytosol and later returned to the primary plastid in the green plant lineage [51-54]. Thus, these α-amylases seem to be an example of the second pathway.

The evolutionary scenario we present for αAmy3, αAmy7, CAH1, and NPP1 also is compatible with the fact that only a small fraction of primary plastid-targeted proteins carry signal peptides. The first analyses of the *A. thaliana* plastid proteome suggested that up to 8% of its proteins could have signal peptides [86,136], but this clearly was an overestimate based on false positive identifications, non-plastid contamination, and envelope proteins with N-terminal transmembrane domains that resemble signal peptides [9,87]. Interestingly, this is comparable to the level of falsely predicted signal peptides in cytosolic and mitochondrial proteins [9,87]. When Zybailov et al. [87] corrected for these factors, they found that signal peptide-carrying proteins represent only 0.6% of the plastid proteome. Moreover, available data indicate that αAmy3, αAmy7, CAH1, and NPP1 are targeted to higher plant plastids via the ES because they need to be glycosylated [26,27,30] and/or dually targeted to both the plastid and cell wall [28,30]. Clearly, these are derived rather than ancestral features for plastid-targeted proteins.

### Evolutionary origin of the endomembrane system-mediated protein targeting to green primary plastids

At present, whether ES-mediated protein targeting evolved early or late in primary plastids is unclear. We can envision two ‘early’ scenarios. In the first, the cyanobacterial ancestor of primary plastids maintained a phagosomal membrane for some time, and it subsequently was transformed into the symbiosome membrane [9,137]. Because biogenesis of the phagosomal/symbiosome membrane would have depended on its fusion with ES-derived vesicles [9], an ES-mediated protein pathway could have originated very early in the evolution of primary plastids. The phagosomal/symbiosome membrane next would have been disrupted by uncoordinated divisions of the cyanobacterial endosymbionts [9], resulting in a chimeric bacterial-eukaryotic outer membrane that still could undergo fusion with ES-derived vesicles. Alternatively, the endosymbiotic cyanobacteria could have escaped from the phagosome very early, not retaining any proteins and lipids from the phagosomal/symbiosome membrane. Comparable escapes have been shown for some intracellular bacteria [138,139]. In this case, the cyanobacterial endosymbionts would have been surrounded by two membranes from the very beginning. Outer membranes of some Gram-negative bacteria form vesicles used in intercellular communication [140,141], and these vesicles could have established vesicular connections between primary plastids and other compartments within the host cell, including ES-mediated protein targeting to primary plastids.

In the ‘late’ scenario, and similarly to the second ‘early’ scenario, the cyanobacterial endosymbiont would have escaped from the phagosome very early, without retention of any proteins and lipids from the phagosomal/symbiosome membrane, Because outer endosymbiont membrane-generated vesicles probably were incompatible with the host system for vesicle formation and fusion, ES-mediated protein targeting to primary plastids would have originated late in their evolution. It is known that the outer membrane of green plant plastids possesses connections with the ER [142-144] and establishment of these connections could have enabled acquisition of eukaryotic lipids and proteins responsible for vesicle formation and fusion by the outer plastid membrane [Bodył et al., manuscript in preparation]. ES-mediated protein targeting to primary plastids could have evolved as a result of this chimerization of the outer membrane.

Although at present we cannot exclude scenarios of an ancient origin of ES-mediated protein import into plastids [9,137], our results clearly favor a ‘late’ model by demonstrating that αAmy3, αAmy7, NPP1, and CAH1 were redirected to green plant plastids fairly recently (Figure 1 B3). Additional support for a late model comes from phospholipid transport to primary plastids. These plastids still uses complete prokaryotic fatty acid and glycerolipid synthesis machinery capable of producing lipids for plastid membranes [145,146]. Therefore, it seems that the original primary plastid did not need to utilize host lipids through fusions with ES vesicles. Although eukaryotic lipid precursors are delivered to modern green plant plastids, this occurs at sites of direct contact between the plastid and ER with the help of bacterial-type TGD proteins [142,147], whereas vesicular transport of lipids has not yet been reported [148]. Moreover, most green algae synthetize only glycerolipids via the prokaryotic pathway, in contrast to higher plants that also or exclusively use the eukaryotic pathway [149-151]. This indicates that the transport of lipid precursors of eukaryotic-type glycerolipids in plastid membranes likely have evolved quite late, perhaps in a correlated manner with ES-mediated protein transport, and certainly well after the primary plastid establishment.

Although all the data and arguments we presented above support a late relocation of αAmy3, αAmy7, NPP1, and CAH1 to primary plastids (Figure 1 B3), it is possible that other, still unidentified, host proteins were imported into primary plastids via ES relatively early (Figure 1 B2). It should be noted that the timing of such relocations is not of the utmost importance to our present model, which postulates that only a small subset of host-derived proteins that already contained signal peptides were directed to primary plastids via ES. Thus, our evolutionary scenario clearly contrasts with the ‘relic’ hypothesis [33], in which all host- and endosymbiont-derived proteins that were targeted to the ancestral primary plastid must have acquired signal peptides and used the ES in their trafficking to the plastid (Figure 1 B1).

### *Paulinella* photosynthetic organelles and the endomembrane system-mediated protein targeting to primary plastids

The amoeba *Paulinella chromatophora* harbors two cyanobacterial endosymbionts/organelles that are deeply integrated with the host cell [152-155]. Bioinformatics analyses suggested that proteins targeted to these two membrane-bound photosynthetic organelles carry N-terminal signal peptides or signal peptide-like domains, suggesting they are imported via the ES [156-158]. This hypothesis recently was confirmed by experimental studies of Nowack and Grossman [159]; therefore, *Paulinella* could be considered a model for the early evolution of the protein import in primary plastids as proposed by Bhattacharya and colleagues [33]. Classic primary plastids and the *Paulinella* organelles evolved independently from distinct cyanobacterial ancestors [154,160,161], however, which suggests they could have been established within their host cells through very different pathways. As evidence of this point, signal peptides characterize proteins targeted to *Paulinella* organelles, including photosynthetic proteins, whereas there is not a single photosynthetic protein with a signal peptide in eukaryotes with classic primary plastids [158,162]. These data imply that the outer membrane of the *Paulinella* organelles could represent the phagosomal membrane derived from the host cell [158,162]. In the case of classic primary plastids, however, the outer membrane clearly has a cyanobacterial origin with eukaryotic components added subsequently, thereby making it chimeric in nature [9,163-166].

Most interestingly, the nuclear and the organelle genomes of *P. chromatophora* both are devoid of genes for Toc75/Omp85 homologs [155,167], meaning their protein products are not available for insertion into the outer endosymbiotic membrane. Why should this be the case? The most reasonable explanation is that ES-mediated transport evolved first; consequently there was no purifying selection to maintain *Toc75/Omp85* genes for protein import into the endosymbiont. Therefore, they decayed and were lost in this endosymbiosis. The pathway by which the *Paulinella* endosymbiosis evolved argues strongly against a ‘relic’ hypothesis for ES-mediated protein transport into classical primary plastids. If ES-mediated transport had come first, there would have been no selective advantage for maintaining Toc and Tic genes, and they would have decayed, just as they have in *Paulinella*.

## Conclusions

Our phylogenetic analyses clearly show that all plastid proteins from *A. thaliana* and *O. sativa*, for which targeting via the ES has been demonstrated experimentally, are of eukaryotic (not cyanobacterial) ancestry. Therefore, their genes were not among those transferred from the cyanobacterial endosymbiont during the early stages of primary plastid evolution. Rather, our results are consistent with a later origin of ES trafficking to primary plastids, possibly only in the higher plant lineage. The ancestors of these few host-derived plastid proteins already were targeted to distinct compartments within the host ES by means of their signal peptides and, therefore, were pre-adapted to be delivered to primary plastids via the same pathway. Moreover, the ES was the only route possible for importing these particular proteins into primary plastids because they require glycosylation and/or are dually targeted to the plastid and the cell wall. We conclude that these proteins constitute a specific group of higher plant plastid-targeted proteins with a peculiar and derived evolutionary history.

## Methods

### Collection of sequences and preparation of alignments

Sequences of homologs to proteins that were proven experimentally to be imported into primary plastids via the endomembrane system were obtained through BLAST searches across several databases: (i) GenBank (non-redundant protein and EST databases [168]), (ii) TbestDB [169], (iii) Dragonblast [170], and (iv) DOE Joint Genome Institute [171]. To verify the BLAST results, and determine domain content of sequences obtained, we searched Conserved Domain Database (CDD) [172].

Amino acid alignments were made in MAFFT version 6.71beta program using slow and accurate algorithm L-INS-i with 1,000 cycles of iterative refinement [173]. The resulting alignments were edited manually in Jalview 2.4.0.b2 [174], and sites suitable for further phylogenetic analyses were extracted from the alignments with Gblocks 0.91b assuming less stringent criteria [175].

### Phylogenetic analyses

Phylogenetic trees were inferred via the Bayesian approach in PhyloBayes 3.2d [176], as well as the maximum likelihood method in PhyMl 3.0 [177] and TreeFinder [178]. For all alignment sets in PhyMl analyses, we applied a LG + I + Γ(5) model of amino acid substitutions as proposed by ProtTest 2.4 [179]. The model was selected assuming optimization of models, branches, and topology of the tree, and considering all criteria (−lnL, AIC, AICc, and BIC). The LG + F + I + Γ (5) model for all alignment sets used in TreeFinder approach was chosen according to the Propose model module in this program assuming optimized frequencies of amino acids and considering all criteria (−lnL, AIC, AICc, BIC, HQ). We applied search depth set to 2 in TreeFinder and the best heuristic search algorithms, NNI and SPR, in PhyMl. Edge support was assessed by bootstrap analyses with 1,000 replicates in each of these two programs. To test alternative tree topologies that considered monophyly of plant and cyanobacterial sequences we used all tests implemented in TreeFinder [178], such as ELW, BP, KH, SH, WSH and AU, assuming 1,000,000 replicates.

We performed two types of analyses in PhyloBayes assuming the model LG + Γ (5) and CAT + Γ (5) with the number of components, weights, and profiles inferred from the data. Two independent Markov chains were run for 100,000 and 1,000,000 cycles for the first and the second approach, respectively. After obtaining convergence, the last 50,000 and 500,000 trees from each chain, respectively, were collected to compute a posterior consensus.

### Prediction of targeting signals and subcellular localization of the analyzed proteins

We applied 20 bioinformatics tools (Table 1) to predict potential N-terminal targeting signals in the sequences analyzed, such as the signal peptide (SP), plastid transit peptide (pTP), and mitochondrial transit peptide (mTP). Appropriate models for prokaryotic and eukaryotic sequences were applied. Sequences in which more than 50% algorithms recognized SP, pTP or mTP were considered to have a given targeting signal and were indicated on the trees presented.

**Table 1:** Programs applied in this study predicting different N-terminal targeting signals

**Program name**	**Reference**
Programs that distinguish SP, pTP and mTP	
iPSORT	[180]
Predotar 1.03	[181]
PredSL	[182]
PProwler 1.2	[183]
TargetP 1.1	[184]
Programs predicting SP	
DetecSig in ConPred II	[185]
HECTAR^SEC^	[186]
Phobius	[187]
PrediSi	[188]
ProtCompB - Version 9	[189]
PSORTb v3.0	[190]
RPSP	[191]
Sigcleave in EMBOSS 3.0.0	[192]
SIGFIND 2.11	[193]
Signal-3 L	[194]
Signal-CF	[195]
SignalP-HMM 3.0	[196]
SignalP-NN 3.0	[197]
SIG-Pred	[198]
SOSUIsignal	[199]

*SP*, signal peptide, *pTP*, plastid transit peptide, *mTP*, mitochondrial transit peptide.

We also searched databases such as The *Arabidopsis* Information Resource (TAIR) [200], The SubCellular Proteomic Database (SUBA) [201], and The WallProtDB database [49] in order to acquire additional information about the localization of proteins analyzed. TAIR maintains genetic and molecular biology data for the model higher plant *A. thaliana*. SUBA houses large scale proteomic and GFP localization sets from cellular compartments of *Arabidopsis* as well as precompiled bioinformatic predictions for protein subcellular localizations. WallProtDB aims at collecting cell wall proteomics experimental results.

## Reviewers’ comments

### Reviewer 1: Prof. Dr William Martin, Institut für Molekulare Evolution, Heinrich-Heine-Universität, Germany

This is an excellent paper. The authors take the theory of Bhattacharya et al. to task, namely, if endomembrane system (ES) mediated targeting was ancestral, then the proteins imported that way should reflect an ancient status. It turns out that if we actually look at the evidence, these few ES mediated imports are of very recent origin, maybe only even in land plants. Thus, the clearest prediction that the Bhattacharya theory makes fails, hence it is not a very robust theory, and if we are honest it doesn’t even account for the proteins upon which it was based. Someone should have noticed this earlier. Gagat et al. put together a scholarly and well-written piece of work, a very welcome addition to the literature. It can be published as is in my view in BD.

Authors’ response: *We are grateful for Prof. Martin’s very positive opinion on our paper, and for all his insightful comments.*

A few comments are: p. 3 is mediated by the oep16 pore is reported to be mediated by the oep16 pore.

Authors’ response: *We modified the corresponding sentence, in accordance with this suggestion.*

The “pioneering” work by Ball had precedent by classical phycologists in the 1970s. The place of starch/polysachharide deposition is a classical character.

Authors’ response: *We modified the sentence in question accordingly and included classical phycologists’ papers about subcellular localization of starch synthesis and degradation in algae.*

p. 18 encouraged us to verify, encouraged us to test.

Authors’ response: *We replaced the word ‘verify’ by ‘test’ and ‘test*’ *by* ‘*check*’ *in the sentence that follows to avoid repetition*.

p. 22, the Buchnera example is problematic because via ES, the vesicles would have to reach the plasma membrane through the cell wall, monomeric lipid import via proteinaceous importers seems more likely.

Authors’ response: *The view that host phospholipids are delivered to Buchnera endosymbionts* (*or bacteriocytes*) *inhabiting insect cells via the vesicular pathway was suggested by Nakabachi et al*. [202]. *This hypothesis is based on the absence of genes encoding enzymes involved in phospholipid biosynthesis in the genomes of these bacterial endosymbionts*[203]. *Because Buchnera endosymbionts with a two*-*membrane envelope reside in symbiosomal vacuoles*, *which are a part of the host ES*, *it is very likely that some vesicles fuse with their surrounding membrane*. *Thus*, *it is reasonable to postulate that these vesicles participate in the transport of host phospholipids to the symbiosomal membrane*, *but their further vesicular trafficking to the endosymbiont*’*s outer and inner membrane is doubtful*, *as was pointed out by the Reviewer*.

*The first stage in the intrasymbiosomal host phospholipid trafficking would be their transport from the symbiosomal to the outer Buchnera membrane*. *Although currently it cannot be excluded that vesicles observed in the lumen of symbiosomal vacuoles are involved in this import step*, *their presence also could result from aging of the bacterium*-*insect endosymbiosis because they were found in older aphids*[204]. *It also could be hypothesized that spontaneous lipid exchange occurs at membrane contact sites between the symbiosomal and the bacterial outer membrane*, *but this was not found in an extensive examination of the envelope membrane system of Buchnera cells*[204]. *Alternatively*, *import could proceed via spontaneous monomeric lipid exchange*, *although this process would be slow and insufficient*[205]. *Thus*, *the most probable trafficking mechanism appears to be import mediated by lipid*-*transfer proteins* (*LTPs*) *that transfer and exchange phospholipids between cellular membranes*[206].

*After insertion into the outer membrane of Buchnera endosymbionts*, *imported host phospholipids would need to move through the periplasmic space containing the peptidoglycan wall to reach the inner endosymbiont membrane*. *The trafficking step across this space remains mysterious*[207,208]. *Nevertheless*, *the peptidoglycan wall is a clear obstacle for vesicle*-*mediate transport*, *as indicated by the Reviewer*. *Consequently*, *some periplasmic carrier proteins*, *rather than vesicles*, *are likely to participate in phospholipid transport*; *however*, *there is no evidence for such proteins in Gram*-*negative bacteria*[208]. *Also phospholipid exchange at membrane contact sites between the outer and inner envelope membranes of Buchnera cells does not appear to be a viable explanation*, *given that a thorough search did not find them*[204].

*A more tractable problem is translocation of phospholipids through the outer and inner Buchnera envelope membranes*, *in which they would rotate* (*or flip*-*flop*) *from the outer to inner leaflet*. *Since phospholipid rotation is thermodynamically unfavorable*, *it is facilitated by flippases*, *for example MsbA*, *a member of the ATP*-*binding cassette* (*ABC*) *transporter family*[209]. *This protein normally is located in the inner bacterial membrane* (*the original site of phospholipid biosynthesis in bacterial cells*) *and in Escherichia coli*, *plays a role in the transport of phospholipids from the inner to the outer leaflet of the inner membrane*[210-212]. *Two homologs of E*.*coli MsbA*, *specifically MdlA and MdlB*, *recently were identified in Buchnera genomes*[204]. *They potentially could participate in phospholipid transport*; *however*, *these transporters are assumed to translocate phospholipids from the inner to outer leaflet*, *whereas in Buchnera cells transport would proceed in the opposite direction*. *Moreover*, *to participate in phospholipid transport in the outer membrane as well*, *they would have to have been relocated there from the inner membrane*. *In contrast to studies on the role of MsbA in phospholipid transport*, *Kol et al*. [213,214]*showed that the translocation of phospholipids is energy independent and may not require a specifically dedicated flippase*, *but merely typical α*-*helical membrane*-*spanning segments of various membrane proteins*. *Thus*, *such translocations would not be specific and selective*. *Similar proteins also could mediate phospholipid transport between membrane leaflets in Buchnera cells*, *which is in agreement with Reviewer*’*s remark*.

*An interesting phospholipid*-*trafficking system that mediates the transport of phospholipids from the outer leaflet of the outer membrane to the inner membrane was discovered in E*. *coli*[215]. *Its function is to maintain lipid asymmetry in the outer membrane and prevent phospholipid accumulation in the outer leaflet of this membrane*. *The system*, *called Mla*, *consists of six proteins*; *an outer membrane lipoprotein*, *a periplasmic substrate binding protein and ABC transporter machinery with four proteins at the inner membrane*. *In this pathway*, *phospholipids are removed from the outer membrane and delivered to the ABC transporter complex via a periplasmic substrate binding protein*. *The system is conserved*, *not only in Gram*-*negative bacteria but also in plant plastids*. *Thus*, *it is possible that a similar system was adapted by Buchnera endosymbionts to import host phospholipids*.

*Regarding the Reviewer*’*s comment and the general controversy concerning phospholipid transport to the envelope membranes of Buchnera endosymbionts*, *we decided to delete the corresponding part of the text from our discussion*. *An additional advantage of our decision to remove this controversial part is that the discussion is now more compact and our ideas flow more clearly*.

p. 23, I would like to see independent confirmation of the Nowack paper, I remain unconvinced. But the present paper does not hinge on that in any way and the authors have earned their say.

Authors’ response: *We completely agree with the Reviewer that further studies on protein import into the photosynthetic endosymbionts*/*organelles of Paulinella chromatophora are necessary*. *The recently published paper by Nowack and Grossman*[159]*represents the first experimental approach to this issue*. *Using immunogold labelling*, *these authors were able to show that three photosynthetic proteins*, *PsaE*, *PsaK1*, *and PsaK2*, *are imported into these photosynthetic entities in the Paulinella strain CCAC0185*. *ES*-*mediated targeting*, *however*, *as suggested previously based on bioinformatic analyses*[156,157], *was demonstrated only for PsaE by Nowack and Grossman*[159]. *Despite that import route*, *this protein does not carry any N*-*terminal signal peptide*, *which generally is used to target proteins to the ES*. *Thus*, *it is possible that the Paulnella PsaE possesses an internal targeting signal*[158]. *In any case*, *how it is translocated into the ER and further trafficked within ES is unknown and needs further investigation*.

*In contrast to PsaE investigated by Nowack and Grossman*[159], *a signal peptide was predicted with high probability in the PsaE protein from another Paulinella strain* (*FK01*) [156]. *Moreover*, *that signal peptide has a clear cleavage site*, *which suggests it is removed in ER*. *Interestingly*, *signal peptide*-*like domains were predicted in four other photosynthetic proteins from the strain CCAC0185*, *PsaK1*, *PsaK2*, *PsbN*, *and the homolog of Synechococcus WH5701*_*13415*[157]. *These data suggest that at least some nuclear*-*encoded proteins are targeted to Paulinella photosynthetic entities via the ES*[155-158].

*No N*-*terminal targeting signals were identified in the four other Paulinella proteins*, *Hli*, *CsoS4A*, *and homologs to Synechococcus WH5701*_*06721 and WH5701*_*13905*[157]. *Considering work on PsaE by Nowack and Grossman*[159], *we could hypothesize that they are targeted via ES*; *however*, *other import routes currently cannot be excluded*. *In that case*, *Paulinella photosynthetic entities would possess several distinct targeting pathways*, *similar to higher plant plastids*[9,20].

*Further experimental studies using other techniques* (*e*.*g*. *pulse*-*chase immunoprecipitation and GFP*-*based methods*) *are needed*, *not only to confirm protein import into Paulinella photosynthetic entities*, *but also to clarify how it occurs*. *The upcoming publication of the Paulinella nuclear genome will provide important new data*. *For example*, *it will be possible to identify additional proteins imported into Paulinella photosynthetic entities*, *and to trace their evolutionary histories*. *Although further experimental studies on protein import into Paulinella photosynthetic entities are ongoing*, *we think they will confirm the results obtained by Nowack and Grossman*[159].

In summary this is a fine paper that evolutionarily inclined readers of BD are going to like.

Quality of written English: Acceptable.

Authors’ response: *Thank you*.

### Reviewer 2: Dr Philippe Deschamps, Unité d’Ecologie, Systématique et Evolution, Université Paris-Sud, France

In this manuscript, Gagat et al. propose to revisit an hypothesis formulated by Bhattacharya et al. in 2007 concerning the early evolution of plastidial protein targeting machineries in Archaeplastida. This hypothesis postulated that, in the very first step of plastid settlement, protein targeting to the plastid was done using the endomembrane network, sending signal peptide tagged proteins, packed into vesicles, to the plastidial compartment. One of the major argument of Bhattacharya et al. was that living green algae and plants still address a couple of proteins to their plastid using the endomembrane system (ES). These remnant cases would represent a relic of an ancient general pathway that used to affect every plastid targeted protein before being replaced by the actual major transit peptide/Tic-Toc pathway.

To challenge this hypothesis, Gagat et al. decided to further study four protein families having unconventionally targeted representatives: α-Amylases, Purple acid phosphatases, α-Carbonic anhydrases and Protein disulfide isomerases.

Their goal was to:

1. Determine if these proteins are of cynobacterial origin. Indeed, endosymbiotic transferred genes are the prime candidates for being rapidly modified to be targeted back the transitional plastid. Proteins inherited from the eukaryotic host have lower chance to be concerned by an early targeting pathway.

2. In the case of an eukaryotic ancestry for the protein family, Check if orthologous proteins of heterotrophic eukaryotes also carry a signal peptide. If so, this is a clue that this tag was not specifically added after primary endosymbiosis for an early targeting purpose.

3. Review the literature to find out if the plastidial location of these proteins is a common trait shared by all Archaeplastida, meaning that this was an early modification that took place in their common ancestor.

The authors could produce phylogenetic trees of all but one protein families. The α-Carbonic anhydrases tree was too much unresolved to be interpreted. The trees presented in figures 4 and 5, respectively acid phosphatases and disulphide isomerases, strongly point to a eukaryotic origin of these proteins in Archaeplastida. Moreover, the secreted nature (via the ES) of purple acid phosphatases studied here clearly predates primary endosymbiosis. Additionally, the unconventional ES plastid targeted disulfide isomerase is a specific feature of Chlamydomonas. Every other similar protein of Chloroplastida do have an additional retention signal preventing the protein from leaving the ER. This retention signal is probably ancestral in the group, demonstrating that ES plastidial targeting is a secondary modification. On the other hand, phylogenetic trees for the interesting subset of apha-amylases are more blurry (Figures 2 and 3) but clearly incompatible with a cyanobacterial origin of these proteins in Archaeplastida. Moreover, the authors clearly show that the use of the ES pathway for some alpha amylases is a secondary modification specific to some land plants and based on ancestral protein that use to have a transit peptide. Finally, despite the impossibility to trace the origin of the ES plastid targeted α-Carbonic anhydrases, the authors provide strong clues that the presence of a signal peptide is a general feature all the proteins related to the 2 sole plastid targeted cases identified in Arabidopsis and Chlamydomonas. These 2 exceptions are clearly late tuning of originally non-plastidial proteins.

Altogether, Gagat et al. present convincing indications that cases of protein unconventionally targeted to the chloroplast of green algae and plants using the endomembrane pathway are probably not residual traces of an ancient general targeting pathway that could have predated the Tic-Toc pathway during the evolution of Archaeplastida. The invalidation of the hypothesis proposed by Bhattacharya et al. encourage to search for a new evolutionary scenario for the development of plastid targeting in Plantae.

I encourage the publication of this article in Biology direct.

Authors’ response: *Thank you for your positive opinion on our paper*.

Nonetheless, I would recommend a small rewriting of the manuscript. The quality of the language is uneven along the text, and some part could be enhanced. Additionally, some typing errors have to be corrected.

Quality of written English: Needs some language corrections before being published.

Authors’ response: *According to this suggestion*, *the whole paper was edited for English usage by Dr*. *John Stiller at East Carolina University*, *NC*, *USA*. *We hope that the Reviewer will be satisfied with the quality of English in our revised article*.

### Reviewer 3: Dr. Simonetta Gribaldo, Unité de Biologie Moléculaire du Gène chez les Extrêmophiles, Department of Microbiology, Institut Pasteur, France

This reviewer provided no comments for publication.

## Abbreviations

αAmy3: α-amylase 3 from *Oryza sativa*; αAmy7: α-amylase 7 from *Oryza sativa*; CAH1: α-carbonic anhydrase 1 from *Arabidopsis thaliana*; CAH3: α-carbonic anhydrase 3 form *Chlamydomonas reinhardtii*; Dsb: Disulfide bond proteins; EGT: Endosymbiotic gene transfer; ES: Endomembrane system; HGT: Horizontal gene transfer; mTP: Mitochondrial transit peptide; NPP1: Nucleotide pyrophosphatase/phosphodiesterase 1 from *Oryza sativa*; NPP3: Nucleotide pyrophosphatase/phosphodiesterase 3 from *Oryza sativa*; PAP: Purple acid phosphatase; PDI: Protein disulfide isomerase; pTP: Plastid transit peptide; RB60: RNA-binding protein 60 from *Chlamydomonas reinhardtii*; SP: Signal peptide.

## Competing interests

The authors declare that they have no competing interests.

## Authors’ contributions

PG carried out phylogenetic and bioinformatics analyses under supervision of PM. AB, PM and PG interpreted the obtained results. PM and AB conceived the studies. PG, AB and PM wrote the manuscript. All authors read and approved the final version of the manuscript.
